# Natural Bioactive Compounds in Polycystic Ovary Syndrome: Properties, Molecular Mechanisms, and Therapeutic Potential

**DOI:** 10.3390/ijms27114715

**Published:** 2026-05-23

**Authors:** Rosa Linares, Gabriela Rosas, Elizabeth Vieyra, Andrea Chaparro, Julieta-Azucena Espinoza, Deyra de los Angeles Ramírez, Carlos-Camilo Silva, Patricia Rosas, Víctor-Manuel Macías, Leticia Morales-Ledesma

**Affiliations:** 1 Endocrinology Laboratory-UIBR, Facultad de Estudios Superiores Zaragoza, Universidad Nacional Autónoma de México, México City 09230, Mexico; 2 Medical Surgeon Career, Facultad de Estudios Superiores Zaragoza, Universidad Nacional Autónoma de México, México City 09230, Mexico; centrofalia@comunidad.unam.mx (C.-C.S.); iram66671@yahoo.es (V.-M.M.); 3 Reproductive Physiology Laboratory-UIBR, Facultad de Estudios Superiores Zaragoza, Universidad Nacional Autónoma de México, México City 09230, Mexico; gabriela_rosasg@comunidad.unam.mx (G.R.); elizabeth_vieyra_valdez@hotmail.com (E.V.); chaparrortegandrea@comunidad.unam.mx (A.C.); azuespinozamoreno@unam.edu (J.-A.E.); 4 Neuroendocrinology of Reproduction Research Laboratory-UIBR, Facultad de Estudios Superiores Zaragoza, Universidad Nacional Autónoma de México, México City 09230, Mexico; 5 Nursing Career, Facultad de Estudios Superiores Zaragoza, Universidad Nacional Autónoma de México, México City 09230, Mexico; 6 Facultad de Estudios Superiores Zaragoza Campus III, Universidad Nacional Autónoma de México, Ex Fábrica de San Manuel 8-A, Comunidad de San Miguel Contla, México City 90640, Mexico; deyra_arh@comunidad.unam.mx; 7 Chronobiology of Reproducción Research Laboratory-UIBR, Facultad de Estudios Superiores Zaragoza, Universidad Nacional Autónoma de México, México City 09230, Mexico; 8 Biology of Reproduction Research Unit, Laboratorio de Neuroinmuno-Endocrinología, Facultad de Estudios Superiores Zaragoza, Universidad Nacional Autónoma de México, Ciudad de México 09230, Mexico; prosas44@yahoo.com.mx

**Keywords:** polycystic ovary syndrome, natural products, bioactive compounds, oxidative stress, insulin resistance, ovarian steroidogenesis

## Abstract

Polycystic ovary syndrome (PCOS) is one of the most common metabolic–endocrine disorders affecting women of reproductive age and represents a significant public health concern due to its clinical heterogeneity. It is characterized by hyperandrogenism, ovulatory dysfunction, and polycystic ovarian morphology, and is frequently associated with hyperinsulinemia, obesity, dyslipidemia, chronic low-grade inflammation, and an increased risk of type 2 diabetes and cardiovascular disease. Conventional treatments, including combined oral contraceptives, metformin, and ovulation-inducing agents, primarily target symptoms and present limitations in efficacy, tolerability, and their ability to address underlying metabolic dysfunction. In this context, naturally derived bioactive compounds have emerged as promising complementary therapeutic strategies. Various phytochemicals exhibit antioxidant, anti-inflammatory, hypoglycemic, and reproductive axis-modulating effects by targeting key molecular pathways involved in insulin resistance, hyperandrogenism, and follicular dysfunction. Emerging preclinical and clinical evidence suggests that these compounds may improve metabolic, hormonal, and reproductive outcomes in women with PCOS.

## 1. Introduction

Polycystic ovary syndrome (PCOS) is one of the most prevalent endocrinopathies affecting women of reproductive age, with an estimated global prevalence ranging from 8% to 13%, although this varies depending on the diagnostic criteria applied and the characteristics of the studied population. Its high prevalence, together with its phenotypic heterogeneity, makes PCOS a major public health concern. According to the Rotterdam criteria established in 2003 and their subsequent refinement in the latest International Evidence-Based Guidelines for the Assessment and Management of PCOS, the syndrome is characterized by the presence of at least two of the following features: clinical and/or biochemical hyperandrogenism, ovulatory dysfunction, and polycystic ovarian morphology [1]. PCOS is also associated with insulin resistance (IR), obesity, dyslipidemia, and an increased risk of developing type 2 diabetes mellitus, cardiovascular disease (CVD), and mood disorders [1,2]. The coexistence of reproductive, metabolic, and psychological disturbances not only complicates clinical management but also increases the health, economic, and social burden of the disease.

Despite advances in understanding its pathophysiology, the management of PCOS remains largely focused on symptom control, with limited therapeutic options targeting the underlying etiological mechanisms. Importantly, current International Evidence-Based Guidelines emphasize lifestyle interventions—including dietary modifications, physical activity, and behavioral strategies—as the first-line approach for managing both the metabolic and reproductive manifestations of PCOS [1,3]. Although lifestyle interventions remain the cornerstone of PCOS management, long-term adherence and efficacy are often variable, highlighting the need for complementary therapeutic strategies that target the underlying metabolic and endocrine dysfunctions [4]. Conventional pharmacologic approaches include combined oral contraceptives to regulate the menstrual cycle and reduce hyperandrogenism, insulin sensitizers such as metformin to improve glycemic homeostasis, and ovulation-inducing agents for women seeking pregnancy, including clomiphene citrate (a selective estrogen receptor modulator) or letrozole (an aromatase inhibitor), each presenting specific limitations in terms of efficacy, safety, and therapeutic scope. These therapies are also limited by adverse side effects, variability in clinical responses, implantation failure, and limited efficacy addressing associated metabolic and cardiovascular comorbidities [1,5].

This review provides a comprehensive analysis of the available scientific evidence regarding the role of selected natural bioactive compounds in the context of PCOS. It examines their main mechanisms of action at the molecular and physiological levels, as well as their effects on the endocrine, metabolic, and ovarian alterations characteristic of the syndrome. The advantages, limitations, and future perspectives of their therapeutic application are discussed, with the aim of highlighting their potential as adjunctive or alternative strategies for the management of PCOS. To this end, a comprehensive literature search was conducted to identify relevant studies investigating the role of natural bioactive compounds in PCOS. The search was performed using Scopus, PubMed/MEDLINE, and Web of Science databases and included publications from January 2000 to March 2025.

The search strategy combined Medical Subject Headings (MeSH; for PubMed) and free-text terms using Boolean operators (AND/OR). The main search terms included the following: “polycystic ovary syndrome” OR “PCOS” AND “bioactive compounds” OR “flavonoids” OR “alkaloids” OR “polyphenols” OR “terpenoids” OR “inositols” OR “phytoestrogens” OR “fatty acids”. Inclusion criteria comprised original research articles (in vitro, in vivo, and clinical studies) evaluating the molecular, metabolic, or reproductive effects of bioactive compounds in PCOS, as well as articles published in peer-reviewed journals. Exclusion criteria included case reports, non-peer-reviewed articles, and studies lacking mechanistic or clinical relevance to PCOS. Study selection was performed through screening of titles, abstracts, and full-text articles.

## 2. Pathophysiology of PCOS

The etiology of PCOS remains incompletely understood; however, it is widely recognized as a multifactorial disorder, involving alterations in the hypothalamic–pituitary–ovarian (HPO) axis, genetic predisposition, metabolic abnormalities, and various environmental influences [6,7]. In addition, several factors may contribute to the persistence and progression of the syndrome, including chronic low-grade inflammation, endothelial dysfunction, leukocytosis [8], oxidative stress, exposure to endocrine-disrupting chemicals, high-calorie diets, sedentary lifestyle, and genetic susceptibility [7]. Prenatal exposure to excess Anti-Müllerian Hormone (AMH), maternal androgens, obesity or hypertension has have also been reported to increase the risk of developing PCOS in offspring [9,10]. Furthermore, emerging perspectives suggest that PCOS may represent an evolutionary “mismatch” disorder, in which modern environmental and lifestyle factors interact with genetic susceptibility, exacerbating its metabolic and endocrine manifestations [11].

Clinically, patients with PCOS commonly present with infertility, menstrual irregularities, acne, hirsutism, seborrheic dermatitis, acanthosis nigricans, alopecia and central obesity [8,10]. The diagnostic criteria have evolved through several consensus statements, culminating in the Rotterdam criteria proposed in 2003 by the European Society of Human Reproduction and Embryology (ESHRE) and the American Society for Reproductive Medicine (ASRM). These criteria, further refined in the latest International Evidence-Based Guidelines for the Assessment and Management of PCOS [1], establish that diagnosis requires the presence of at least two of the following features, after exclusion of related disorders: clinical and/or biochemical hyperandrogenism, ovulatory dysfunction, and polycystic ovarian morphology. Based on these criteria, four phenotypes of PCOS are recognized: (A) hyperandrogenism, ovulatory dysfunction, and polycystic ovarian morphology; (B) hyperandrogenism and ovulatory dysfunction; (C) hyperandrogenism and polycystic ovarian morphology; and (D) ovulatory dysfunction and polycystic ovarian morphology [7,12].

At the neuroendocrine level, PCOS has been proposed to originate from an abnormal increase in the frequency of gonadotropin-releasing hormone (GnRH) pulses, which favors luteinizing hormone (LH) secretion over follicle-stimulating hormone (FSH) secretion [12,13]. In the ovary, this altered gonadotropin pattern promotes an increase in the number of antral follicles, premature activation of primordial follicles, and enhanced steroidogenesis [9]. High LH concentrations also induce the hyperactivity of theca cells, which overexpress steroidogenic enzymes, particularly cytochrome P450c17, resulting in increased androgen production. The steroidogenic activity of theca cells is mediated by LH receptors, which are hypomethylated in PCOS, leading to increased gene expression and enhanced sensitivity to LH [12]. Furthermore, insulin promotes ovarian follicular growth and hormone secretion through the activation of its receptors on follicular cells, while also stimulating ovarian steroidogenesis via the activation of the P450c17 and P450scc enzymes, with LH exerting a synergistic effect [14]. The dysregulation of enzymes such as 3β-HSD and 17β-HSD further promotes testosterone accumulation, whereas reduced sulfation increases active androgen levels [15,16,17]. In addition, hyperinsulinemia has been associated with reduced aromatase activity in granulosa cells, impairing the conversion of androgens to estrogens and thereby contributing to androgen excess and follicular dysfunction in PCOS [18].

Hyperandrogenism is a key feature in the pathophysiology of PCOS. When free testosterone levels are not elevated, androstenedione and dehydroepiandrosterone sulfate (DHEAS) levels should also be evaluated. It is essential to exclude other causes of hyperandrogenism, including Cushing’s syndrome, congenital adrenal hyperplasia, hyperprolactinemia, ovarian tumors, and IR [1,10]. Both testosterone and androstenedione can be converted into estrogens by aromatase in granulosa cells. However, in PCOS, aromatase activity and/or expression is frequently reduced, partly influenced by hyperinsulinemia, leading to impaired androgen-to-estrogen conversion and thereby contributing to hyperandrogenism and relatively low or normal estradiol levels [18]. In addition, decreased progesterone levels in PCOS are primarily a consequence of chronic anovulation rather than a direct effect of altered aromatase activity [19]. Polycystic ovarian morphology is typically assessed by transvaginal ultrasonography and is characterized by the presence, in at least one ovary, of more than 20 preovulatory or antral follicles (i.e., measuring 2–9 mm in diameter) and/or increased ovarian volume (>10 mL) [20].

## 3. Pathophysiology of PCOS and Its Interaction with Insulin Resistance, Low-Grade Chronic Inflammation, and Mitochondrial Oxidative Stress

### 3.1. Insulin Resistance

IR is one of the most frequent metabolic abnormalities associated with PCOS, affecting up to 75% of women with the syndrome [21]. Insulin sensitivity is a continuous variable, whereas IR is typically defined as a categorical variable based on specific cut-off values derived from methods such as the euglycemic–hyperinsulinemic clamp (e.g., glucose disposal rate < 4.45 mg/kg/min) or surrogate indices such as the homeostatic model assessment of insulin resistance (HOMA-IR) [21,22]. Both insulin sensitivity and the prevalence of IR may vary among PCOS phenotypes, with some phenotypes, particularly phenotype D, generally exhibiting greater insulin sensitivity and a lower prevalence of IR [21].

Persistent hyperinsulinemia may act as both a cause and a consequence of hyperandrogenism by increasing the activity of the P450c17 enzyme in theca cells and promoting an ovarian environment that favors the formation of multiple small follicles and persistence of antral anovulatory follicles [21]. Approximately 70–75% of patients develop metabolic and cardiovascular comorbidities, including type 2 diabetes, dyslipidemia, hypertension, and an increased risk of endometrial hyperplasia and cancer [19,23]. Hyperandrogenism is also associated with endothelial dysfunction and an elevated risk of cardiovascular disease (CVD) [24]. Indeed, PCOS has been strongly associated with cardiovascular events, such as ischemic heart disease, stroke, atrial fibrillation, and diabetes, particularly in premenopausal women [25]. The development of CVD in women with PCOS is multifactorial and involves genetic and epigenetic factors, oxidative stress, inflammation, and environmental influences. Nevertheless, excess androgen levels and hyperinsulinemia are considered major contributors to cardiovascular risk in these patients [24]. Women with PCOS and IR also exhibit a significantly increased risk of pregnancy loss and chronic metabolic disorders, including type 2 diabetes mellitus, cardiovascular disease, and metabolic syndrome [21].

Insulin is secreted by pancreatic β cells and binds to its receptors located on the cell membrane. The insulin receptor is a heterotetramer composed of α and β subunits linked by disulfide bonds. The extracellular α subunits are responsible for ligand binding, whereas the β subunits are transmembrane glycoproteins with intrinsic tyrosine kinase activity. Insulin binding induces receptor autophosphorylation in tyrosine residues, leading to the phosphorylation of insulin receptor substrates 1-4 (IRS1-4), and triggering a complex intracellular cascade that initiates insulin signal transduction [21].

One of the major insulin signaling pathways is mediated by the serine/threonine kinase (Akt)/protein kinase B (PKB), also known as the phosphatidylinositol 3-kinase (PI3-K) pathway. Through this pathway, insulin stimulates glucose uptake by promoting the translocation of glucose transporter type 4 (GLUT4) from intracellular vesicles to the plasma membrane. In addition, insulin signaling promotes the inhibition of glycogen synthase kinase 3 (GSK3), thereby increasing the synthesis of glycogen, fatty acids, and proteins. Alterations characterized by increased serine phosphorylation and reduced tyrosine phosphorylation of insulin receptors can impair insulin signal transduction, contributing to insulin resistance in women with PCOS [21]. Given its central role in the pathophysiology of PCOS, IR represents an important therapeutic target for various natural bioactive compounds.

### 3.2. Chronic Low-Grade Inflammation

Another important factor associated with PCOS is chronic low-grade inflammation, which may contribute to anovulation, impaired embryo implantation, and infertility [10]. Obese women with PCOS have been reported to exhibit a pro-inflammatory state characterized by increased circulating leukocytes and immune cells, including macrophages, lymphocytes, and eosinophils, as well as high levels of C-reactive protein (CRP) [26], interleukin 1-β (IL-1β), and interleukin 17 (IL-17) [6]. The inflammatory response has also been linked to hyperandrogenism, as increased androgen levels can stimulate monocyte infiltration into the ovary and increase the production of interleukin-6 (IL-6) and tumor necrosis factor-α (TNF-α), thereby altering ovarian follicular dynamics and promoting the development of follicular cysts [8].

In patients with PCOS, adipocytes within visceral fat undergo hypoxia-induced necrosis, promoting the recruitment of inflammatory cells and the production of multiple pro-inflammatory cytokines that sustain chronic low-grade inflammation. As a consequence, oocyte development may be impaired and ovulation might be disrupted [10]. In addition, chronic low-grade inflammation in PCOS has been associated with alterations in the gut microbiota. Gut dysbiosis can increase intestinal permeability and promote metabolic endotoxemia, thereby contributing to systemic inflammation and metabolic disfunction [27]. Therefore, chronic inflammation represents a key mechanism in PCOS and a therapeutic target for natural bioactive compounds with anti-inflammatory properties.

### 3.3. Oxidative Stress and Mitochondrial Dysfunction

Several studies point to oxidative stress and mitochondrial dysfunction as key factors in the onset, progression, and pathophysiology of PCOS [23,28]. Reactive oxygen species (ROS) activate intracellular signaling cascades such as p38 mitogen-activated protein kinase (p38 MAPK) and protein kinase C (PKC), which in turn modulate transcription factors involved in steroidogenesis. ROS can influence transcription factors such as CREB and AP-1 (FOS/JUN), which participate in the regulation of steroidogenic enzymes, including cytochrome P450 family 17 subfamily A member 1 (CYP17A1). In vitro studies using steroidogenic cell models, including ovarian theca and granulosa cells, have demonstrated that oxidative stress alters MAPK- and PKC-dependent signaling pathways, leading to changes in transcription factor activity and downstream steroidogenic gene expression. These findings suggest that CYP17A1 may be indirectly regulated under oxidative conditions. Collectively, this mechanistic framework provides a biologically plausible link between mitochondrial dysfunction, oxidative stress, and increased androgen production in PCOS [29,30,31,32].

Oxidative stress in PCOS is influenced by multiple nutritional, environmental, and endogenous factors. Nutritional factors such as high-calorie diets, increased intake of saturated fats, and low antioxidant consumption may promote excessive ROS generation. Environmental exposures, including endocrine-disrupting chemicals and pollutants, have also been associated with increased oxidative stress. In addition, endogenous metabolic disturbances such as obesity, hyperinsulinemia, chronic inflammation, and mitochondrial dysfunction further contribute to ROS overproduction and impaired antioxidant defense systems, thereby exacerbating oxidative stress in PCOS [33,34]. ROS include the superoxide anion (O_2_^−^), hydrogen peroxide (H_2_O_2_), and hydroxyl radicals (-OH) which are generated as natural byproducts of aerobic metabolism. Among these species, O_2_^−^ and H_2_O_2_ are the more stable and biologically relevant because they can act as signaling molecules produced in response to diverse environmental and hormonal stimuli, thereby functioning as cellular second messengers [34,35]. ROS also participate in several physiologic processes, including the regulation of mitochondrial turnover and mitophagy. In skeletal muscle, increased H_2_O_2_ production has been associated with enhanced glucose uptake and metabolism. In addition, ROS generated in the macula densa cells are critical mediators of tubuloglomerular feedback, contributing to the regulation of glomerular hemodynamics and filtration [36].

ROS are primarily generated by the mitochondrial electron transport chain and NADPH oxidase. Electron leakage at Complex I (flavoprotein domain) and Complex III (semiquinone radical binding site) results in the formation of O_2_^−^ [25,26]. Under physiological conditions, antioxidant defenses such as superoxide dismutase (SOD) and glutathione peroxidase (GPx) maintain redox homeostasis. However, when ROS production exceeds antioxidant capacity, oxidative stress occurs [37,38].

In PCOS, both endocrine and metabolic risk factors have been associated with ROS overproduction, particularly through mitochondrial abnormalities [28,39]. Dysfunctional mitochondria generate elevated levels of ROS within the electron transport chain, thereby contributing substantially to oxidative stress [15]. Increased ROS levels and lipid peroxidation markers such as malondialdehyde (MDA), have been detected in the serum and follicular fluid of patients with PCOS [28,40]. When ROS production exceeds the capacity of antioxidant defense systems, oxidative stress damages lipids, proteins, and DNA, which as reflected by the increase in the MDA [37]. In PCOS, oxidative damage exacerbated by mitochondrial dysfunction, reduces the activity of antioxidant enzymes such as manganese SOD and GPx, leading to ROS accumulation. This contributes to the ovarian dysfunction observed in PCOS by interfering with oocyte maturation and steroidogenesis [15,41,42].

ROS plays an important role in modulating androgen synthesis and metabolism by influencing the expression and activity of rate-limiting enzymes involved in steroidogenesis. Oxidative stress has been associated with increased CYP17A1 activity, which converts pregnenolone and progesterone into androstenedione and dehydroepiandrosterone (DHEA), through signaling pathways such as Protein-Associated Kinase (MAPK) [17]. ROS may also alter the activity of 3β-hydroxysteroid dehydrogenase (3β-HSD) and 17β-hydroxysteroid dehydrogenase (17β-HSD), enzymes involved in the conversion of DHEA to androstenedione and subsequently testosterone. In addition, oxidative stress may inhibit sulfotransferase (SULT) enzymes, which normally sulfate and inactivate androgens, thereby increasing levels of bioactive androgens [17]. Collectively, these alterations contribute to the hyperandrogenism characteristic of PCOS.

ROSs are also involved in follicular development and ovulation. Under physiological conditions, ROS function as second messengers that activate signaling pathways such as MAPK to promote granulosa cell proliferation and supporting normal ovulation [34,35]. However, excessive ROS levels induce oxidative damage in granulosa cells by compromising mitochondrial integrity, altering mitochondrial membrane potential, and promoting opening of the mitochondrial permeability transition pore. This process leads to the release of cytochrome C into the citosol, which consequently activates caspase-9 and caspase-3. In parallel, ROS-induced DNA damage stabilizes p53, shifts Bax/Bcl-2 balance towards apoptosis [42], and upregulates Fas/FasL signaling [41]. Simultaneously, activation of matrix metalloproteinases contributes to extracellular matrix degradation c, affecting follicular architecture and oocyte quality [15]. Collectively, these mechanisms highlight oxidative stress and mitochondrial dysfunction as central drivers of ovarian dysfunction in PCOS and support their relevance as promising therapeutic targets for natural bioactive compounds.

## 4. Traditional Treatments for PCOS

Conventional treatment strategies for PCOS are based on lifestyle modifications and pharmacological interventions aimed at managing its endocrine and metabolic symptoms. These approaches include oral contraceptives to regulate the menstrual cycle and reduce androgen levels, whereas metformin is commonly used to improve insulin sensitivity. In patients with infertility, ovulation-inducing drugs such as clomiphene citrate or letrozole are prescribed [2,43,44,45].

Although metformin reduces hyperinsulinemia, it is not considered a first-line agent for ovulation induction in women with PCOS, as it has shown lower efficacy than other fertility treatments, such as clomiphene citrate or letrozole [43]. Nevertheless, clinical evidence suggests that metformin is a useful therapeutic option in women with PCOS and metabolic disorders because it improves lipid and glycemic profiles. However, its use is associated with gastrointestinal side effects, including nausea or diarrhea. In addition, prolonged treatment with metformin has been linked with vitamin B12 deficiency, and its use is contraindicated or requires caution in patients with impaired renal function because of the risk of drug accumulation and lactic acidosis [46].

Combined oral contraceptives are among the most widely used therapies for the management of menstrual irregularities and hyperandrogenism in PCOS. These agents suppress gonadotropin secretion and reduce ovarian androgen production, gradually improving acne and hirsutism [2]. However, their therapeutic effects are primarily symptomatic, as they do not correct IR or the underlying metabolic abnormalities associated with the syndrome [5]. Furthermore, their use is associated with formulation-dependent adverse effects, including alterations in carbohydrate and lipid metabolism, an increased risk of thromboembolic events, and contraindications in women with obesity or cardiovascular risk factors. Therefore, their selection should be individualized according to each patient’s risk profile and therapeutic goals [2].

Clomiphene citrate, a selective estrogen receptor modulator, has historically been used as a first-line treatment for ovulation induction in women with PCOS seeking pregnancy. However, its efficacy may be limited in some patients, and compared to aromatase inhibitors such as letrozole, it is associated with lower reproductive success and live birth rates [44,47]. Furthermore, Clomiphene citrate may increase the risk of multiple pregnancies and produce adverse effects such as hot flashes or visual disturbances. Moreover, it does not improve the metabolic or hyperandrogenic symptoms of the syndrome.

In recent years, letrozole has emerged as an effective alternative for ovulation induction in PCOS patients. Its mechanism involves reducing the peripheral conversion of androgens to estrogens, thereby decreasing negative feedback at the hypothalamic–pituitary level, and increasing FSH secretion, which promotes follicular maturation. Compared with Clomiphene citrate, Letrozole is associated with a lower risk of multiple pregnancy and reduced antiestrogenic effects on the endometrium and cervical mucus [48,49]. These characteristics have led several clinical guidelines to recommend Letrozole as a first-line treatment for PCOS-related anovulation [45]. However, letrozole also presents certain limitations. Its efficacy may be reduced in women with obesity, severe IR, or extreme hyperandrogenism because metabolic and endocrine alterations can impair the follicular function. In addition, variations in optimal dosing regimens and administration protocols complicate treatment standardization [50]. Although current evidence does not indicate an increased risk of congenital malformations, concern persists among clinicians because Letrozole remains an off-label treatment in some countries. Finally, not all patients respond adequately, and some require combination therapy with metformin or gonadotropins. Lifestyle interventions, based on diet, exercise, and weight control, remain the cornerstone of comprehensive PCOS management [1].

Overall, current treatments for PCOS present significant limitations because they only partially address the heterogeneous phenotypes of the syndrome. Metformin offers moderate metabolic benefits; combined oral contraceptives help regulate the menstrual dysfunction and hyperandrogenism, but do not correct the endocrine and metabolic abnormalities; and ovulation induction therapies improve fertility but do not improve other manifestations of the disorder [1]. In this context, naturally occurring bioactive compounds have attracted increasing interest as potential therapeutic and adjunctive agents for the management of PCOS. Several preclinical and clinical studies have demonstrated that phytochemicals such as flavonoids, polyphenols, phytoestrogens, terpenoids, and alkaloids possess antioxidant, anti-inflammatory, hypoglycemic, and modulatory effects on the neuroendocrine–reproductive axis. These compounds not only improve specific clinical manifestations but also target key molecular mechanisms involved in IR, oxidative stress, chronic low-grade inflammation, and dysfunctional steroidogenesis. Therefore, the systematic study of natural bioactive compounds represents an emerging therapeutic strategy with the potential to complement and broaden current approaches for PCOS treatment by targeting fundamental pathogenic mechanisms of the syndrome.

## 5. Bioactive Natural Products and Mechanisms of Action

**Figure 1:**
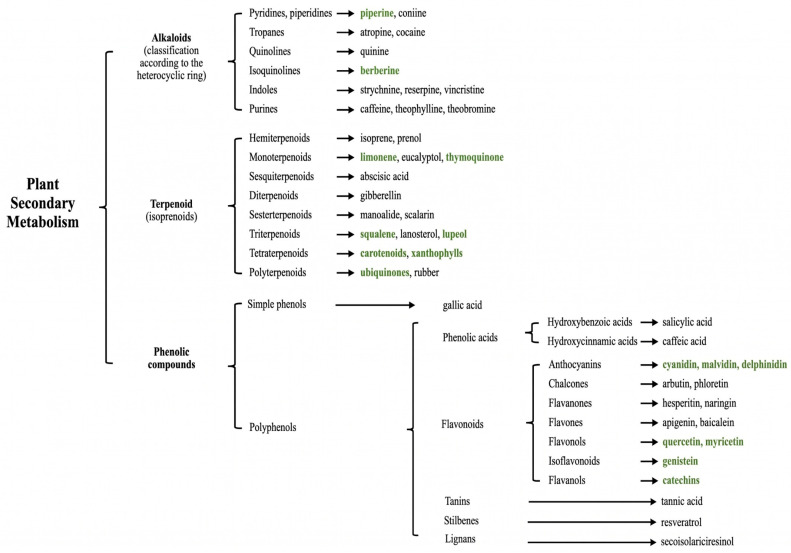
General summary of the classification of plant secondary metabolites. The conventional triad (alkaloids, terpenoids and phenolic compounds) is shown. Some of the secondary metabolites involved in modulating the PCOS and analyzed in this review are highlighted in green.

Plants synthesize a wide variety of secondary metabolites, also known as bioactive compounds, phytochemicals, natural products, or plant constituents, which form the basis of many commercial drugs, as well as herbal remedies derived from medicinal plants. Their classification is based on chemical structure, composition, solubility in various solvents, or biosynthetic pathway. An immense diversity of secondary metabolites arises from the major metabolic pathways of nitrogen, lipids and carbohydrates through specialized biosynthetic processes, giving rise to three main groups: terpenoids, alkaloids and phenolic compounds (Figure 1). These chemical constituents possess biological activities that can improve human health [51].

Bioactive compounds exert differential effects on the phosphatidylinositol 3-kinase (PI3K)/Akt signaling pathway by targeting distinct molecular nodes within the insulin signaling cascade. Inositols, particularly myo-inositol and D-chiro-inositol, enhance insulin sensitivity by improving insulin receptor signaling and promoting insulin receptor substrate 1 (IRS-1) activation, thereby facilitating downstream PI3K/Akt signaling and GLUT4 translocation [52,53]. Polyphenols such as resveratrol have been shown in vitro to restore insulin signaling through activation of adenosine monophosphate-activated protein kinase (AMPK) and improvement in PI3K/Akt pathway activity in insulin-resistant adipocytes [54]. Additionally, oxidative stress has been shown to impair PI3K/Akt signaling in ovarian granulosa cells, suggesting that this pathway is sensitive to redox imbalance in reproductive tissues [55]. Fatty acids, including omega-3 polyunsaturated fatty acids, have also been reported to improve metabolic parameters and insulin sensitivity [56].

Importantly, the efficacy of these compounds may vary across different clinical presentations of PCOS, as defined by current international evidence-based guidelines [1]. Although phenotypic classifications (commonly described as phenotypes A–D) remain useful for research purposes, current evidence emphasizes variability in metabolic risk profiles. In this context, patients presenting with hyperandrogenism and IR (corresponding to phenotypes A and B) exhibit more pronounced alterations in insulin signaling pathways, including PI3K/Akt dysfunction [57]. In contrast, individuals with ovulatory PCOS or without hyperandrogenism (often referred to as phenotypes C and D) tend to exhibit milder metabolic disturbances, suggesting that bioactive compounds targeting oxidative stress and inflammation may exert indirect beneficial effects on insulin signaling in these groups. Therefore, insulin-sensitizing compounds such as inositols and omega-3 fatty acids may be particularly relevant in hyperandrogenic and insulin-resistant phenotypes, whereas compounds with predominant antioxidant and anti-inflammatory properties, including several polyphenols, may provide complementary benefits in phenotypes characterized by milder metabolic dysfunction.

### 5.1. Alkaloids

Alkaloids are a broad group of naturally occurring compounds that account for nearly 20% of plant secondary metabolites [58,59]. These molecules are generally low-molecular-weight organic compounds with alkaline properties, commonly characterized by the presence of at least one nitrogen-containing heterocyclic ring. In their purified form, many alkaloids are crystalline, relatively stable, and colorless [58,60]. A wide variety of biological activities have been attributed to alkaloids, including antibacterial, antifungal antiviral [61], anesthetic, stimulant, and anticancer effects [62]. Their structural diversity arises from the different combinations and arrangements of functional groups synthesized by plants, animals [61,62,63], fungi, and bacteria [62,63]. Among plants, alkaloids are especially abundant in the families Berberidaceae, Amaryllidaceae, Liliaceae, Leguminaceae, Papaveraceae, Ranunculaceae, and Solanaceae [61,64].

Because of their marked structural diversity, alkaloids do not have a universally accepted classification [65]. Plant alkaloids can be divided into different classes depending on their chemical structure, biosynthetic origin, or their taxonomic distribution within the plant kingdom [58,61,66,67]. Among these approaches, classification based on chemical structure is the most commonly used [63]. Under this criterion, alkaloids are generally divided into heterocyclic and non-heterocyclic compounds depending on the position of the nitrogen atom in their chemical structure [61,63]. Based on their biosynthetic origin, alkaloids can be divided into true alkaloids (heterocyclic), protoalkaloids (non-heterocyclic), and pseudoalkaloids. True alkaloids and protoalkaloids are derived from amino acids, while pseudoalkaloids are generally derived from acetate, pyruvic acid, adenine/guanine, or geraniol [63,66,68,69]. Finally, alkaloids produced by plant species of the same genus are grouped together [61,67].

Among true alkaloids, isoquinoline alkaloids constitute one of the largest and most widely distributed groups in the plant kingdom [63,70]. These compounds are characterized by the presence of isoquinoline or tetrahydroisoquinoline rings in their molecules, which are derived from phenylalanine and tyrosine. An important category within this groups comprises protoberberines, which represent 25% of all known isoquinoline alkaloid structures, making them the most common nitrogenous secondary metabolites in nature [71]. Representative protoberberines include palmatin [72], jatrorrhizin [73], magnoflorin [74] and berberine [75].

Piperidine alkaloids constitute another important class of true alkaloids. These compounds are biosynthesized from L-lysine and their structure contains a ring of six radicals, five methylene groups, and one amine group. Piperine is one of the best-known representatives of this class and together with berberine, is among the alkaloids most extensively studied for the treatment of PCOS [63,67,76,77]. Piperine is an amide alkaloid with pungent aroma, found in the fruits of several species of the Piperaceae family, such as *Piper nigrum* L. (black pepper), *Piper longum* L. (long pepper) [78] and *Piper retrofractum Vahl* (Balinese long pepper) [79]. The therapeutic effects of piperine are derived from its anti-inflammatory, antioxidant, immunomodulatory, and antitumor properties [80,81,82]. In addition, increasing evidence suggests that piperine improves metabolic health and weight management, supporting its potential role as a promising natural remedy for diabetes management and PCOS treatment [83].

Computational approaches have been used to evaluate the interaction of piperine and genes involved in the pathogenesis of PCOS, particularly those related to hyperandrogenism and oligomenorrhea. The genes that encode for the following receptors were analyzed: group C nuclear receptor subfamily 1 member (NR3C1), peroxisome proliferators activated receptor gamma (PPAR-γ), transcription factor AP-1 subunit C-Fos, CYP17A1 and glucose 1-dehydrogenase/hexose 6-phosphate dehydrogenase (H6PD). Molecular docking analysis revealed that piperine has the highest binding affinity for the PPAR-γ receptor (−8.34 kcal/mol) and H6PD (−8.70 kcal/mol). These interaction patterns suggest a potential for piperine to modulate these receptors (piperine-protein interactions) [83].

The expression of PPAR-γ in the ovary has been associated with follicle formation and normal ovarian function. Likewise, H6PD has been implicated in several metabolic pathways altered in PCOS such as glucose metabolism, oxidative stress, steroidogenesis, insulin sensitivity, and lipid metabolism [69]. Based on this, Francis et al. (2024) suggest that a computational approach combined with experimental studies may provide a useful framework for the development of targeted therapies for PCOS and related endocrine disorders [83].

In a study by Prasad et al. (2023), rats were fed a high-fat diet for 60 days and subsequently treated with piperine (40 mg/kg body weight) for one month [77]. Piperine administration significantly improved metabolic parameters, antioxidant enzyme activity, carbohydrate metabolism enzymes, and the expression of genes encoding signaling proteins for insulin. Furthermore, molecular docking analysis revealed strong binding of piperine to key regulatory proteins related to diabetes and IR, including protein kinase B (Akt) (−6.2 kcal/mol), the insulin receptor (−7.02 kcal/mol), the key substrate of IRS-1 (−6.86 kcal/mol), GLUT-4 (−6.24 kcal/mol), the substrate Akt 160kD (AS160) (−6.28 kcal/mol), and β-arrestin (−6.01 kcal/mol). Based on these findings, authors proposed that piperine may improve glucose homoeostasis by activating the insulin receptor/IRS-1/Akt/β-arrestin/GLUT4 pathway, enhancing insulin sensitivity and glucose uptake in the high-fat diet-induced type 2 diabetes mellitus model [77].

Berberine is an alkaloid with a wide range of pharmacological activities that has been isolated from several medicinal plants, including *Berberis vulgaris*, *Berberis aristata*, *Berberis aquifolium*, *Coptis chinensis*, *Hydrastis Canadensis* [84,85,86]. Berberine-containing formulations are currently available in both patented preparations [85,87,88] and dietary supplements [89]. Traditionally, berberine has been used in the treatment of dysentery, diarrhea [90], inflammation, diabetes, obesity, eye conditions, cardiovascular, respiratory, and infectious diseases [91,92]. Studies using rodent models of PCOS have shown that oral administration of berberine improves the metabolic, hormonal, and reproductive alterations in a dose-dependent manner (Table 1).

A computational analysis employing virtual docking simulations evaluated the interaction of several isoquinoline alkaloids obtained from *Tinospora cordifolia* stems (palmatine, jatrorrhizin, magnoflorine and berberine) with the human androgen receptor (1E3G). The results showed that berberine (−8.23 kcal/mol) and palmitine (−6.71 kcal/mol) have the highest binding affinity for the active site of 1E3G. These findings suggest that both alkaloids may act as potential antagonists of androgen receptor signaling, supporting their possible therapeutic relevance in PCOS [99].

The roles of berberine and metformin in the treatment of PCOS is a topic that is still being investigated. Metformin has consistently been shown to improve insulin sensitivity and reduce circulating glucose levels, which may contribute to regulate the menstrual cycle. In some patients, metformin promotes ovulation, although its efficacy as monotherapy is variable [100]. Because of its well-stablished metabolic benefits, metformin is widely recommended inclusion in clinical guidelines for the treatment of PCOS, particularly in patients who are overweight or at metabolic risk [101]. Berberine likewise exerts important effects on IR. Several studies report improvements in lipid profile and ovulatory function, with results comparable to those of metformin in some patients [102,103]. Berberine also improves hormonal imbalances by reducing testosterone levels, increasing of sex hormone-binding globulin (SHBG), and alleviating the clinical symptoms of hyperandrogenism, such as hirsutism and acne. Evidence also suggests that berberine may enhance the therapeutic efficacy of other treatments commonly used in PCOS, such as metformin and oral contraceptives [104]. A meta-analysis of randomized controlled trials reported that daily administration of 1500 mg of berberine reduces glycated hemoglobin (HbA1c) by approximately 0.9%, an effect comparable to that achieved by standard doses of metformin (1.0–1.1%) [105]. Moreover, both berberine and metformin have been shown to be equally effective in treating hyperandrogenism and hyperinsulinemia in women with PCOS undergoing in vitro fertilization (IVF) [106].

In clinical trials, patients with PCOS and IR treated with berberine hydrochloride (500 mg three times daily for three months) showed a decrease in waist circumference and waist-to-hip ratio (WHR), total cholesterol (TC), triglycerides (TG), and low-density lipoprotein cholesterol (LDLC), as well as an increase in high-density lipoprotein cholesterol (HDLC), and SHBG, as compared to those treated with metformin. Although both berberine and metformin improved glycemic parameters, their effects on lipid metabolism was different. The improvement in lipid dysregulation induced by berberine was greater than that obtained with metformin. These results suggest that berberine intake improves metabolic and hormonal disorders in women with PCOS, where the main effects may be related to changes in body composition associated with obesity and dyslipidemia [102]. Furthermore, administration of a lower dose of berberine (400 mg three times daily for four months) to women with PCOS improved the menstrual pattern and ovulation rate, and reduced SHBG, IR, TC, TG, and LDLC [103].

Later Rondanelli et al. [88] showed the effectiveness of treatment with berberine phospholipid (Berberine Phytosome, berberine phospholipids/PRO, Berbevis, patent WO2019/150225; 550 mg/tablet 2 tablets a day for 60 days) To decrease IR assessed by the HOMA-IR. In addition, a decrease in inflammation was observed using CRP, TNF-α, TG, testosterone, body mass index (BMI), visceral adipose tissue (VAT), and scores on symptoms correlated with hyperandrogenism, such as acne, using the Acne Global Classification System (GAGS) and the Cardiff Acne Disability Index (CADI), and body composition using dual- energy X- ray DXA absorptiometry. Therefore, berberine may represent a safe dietary supplement, useful as a therapeutic strategy for women with PCOS [88].

Patients with PCOS undergoing IVF procedures after three months of treatment with berberine hydrate or metformin (500 mg three times a day, orally) showed an increase in the pregnancy rate (berberine 18 (48.6%) vs. metformin 14 (36.8%)) and a reduction in the incidence of severe ovarian hyperstimulation syndrome (OHSS). In this sense, berberine showed a more pronounced therapeutic effect and achieved more live births with fewer side effects than metformin [106].

**Figure 2:**
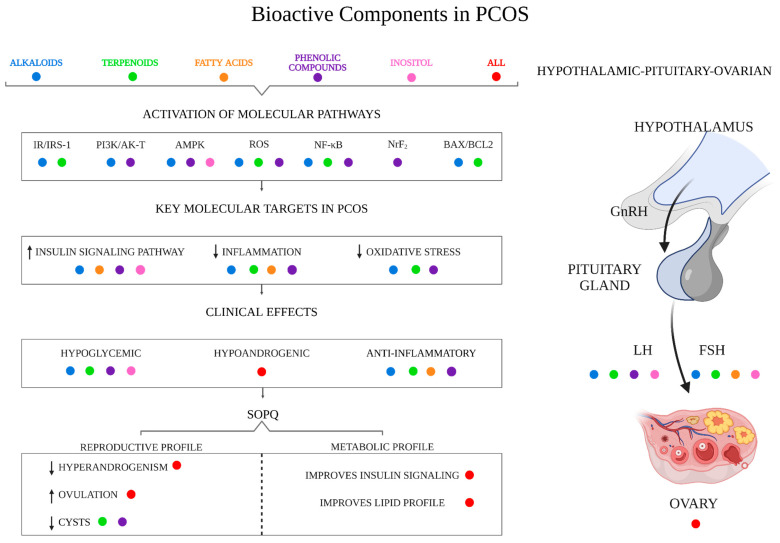
Proposed molecular and physiological mechanisms of bioactive compounds in polycystic ovary syndrome (PCOS). Bioactive compounds, including alkaloids, terpenoids, fatty acids, phenolic compounds, and inositol, modulate key molecular targets associated with PCOS pathophysiology. These compounds regulate signaling pathways involved in insulin sensitivity, inflammation, and oxidative stress, including IR/IRS-1, PI3K/AKT, AMPK, ROS, NF-κB, Nrf2, and BAX/BCL2 pathways. Through these mechanisms, bioactive compounds exert hypoglycemic, hypoandrogenic, and anti-inflammatory effects, contributing to improvements in reproductive and metabolic dysfunctions associated with PCOS, such as hyperandrogenism, ovulatory alterations, ovarian cyst formation, insulin resistance, and dyslipidemia. Additionally, these effects may influence hypothalamic–pituitary–ovarian axis regulation by modulating gonadotropin secretion and ovarian function. AKT, threonine kinase; AMPK, adenosine monophosphate-activated protein kinase; FSH, Follicle-Stimulating Hormone; GnRH, Gonadotropin-Releasing Hormone; IR, insulin resistance; IRS-1, insulin receptor substrate 1; LH, Luteinizing Hormone; NF-κB, nuclear factor kappaB; Nrf2, Nuclear factor erythroid 2-related factor 2; PI3K, phosphatidylinositol 3-kinase; ROS, Reactive oxygen species. Each colored dot represents a bioactive component. Downward arrows indicate decreases, and upward arrows indicate increases.

Kuang et al. [107] showed that berberine administration (100 μM/24 h) to granulosa cell cultures from infertile women with PCOS increases cellular glucose uptake and improves glucose tolerance, while simultaneously increasing insulin sensitivity. This leads to reduced blood glucose levels and improved IR [107]. Meanwhile, berberine at a dose of 50 μM (24 h) decreases the expression of genes associated with inflammation (TLR4, MyD88, monocyte, MCP-1, IL-1β, and IL-6), as well as hyaluronan synthase 2 (HAS2) and therefore hyaluronan synthesis [97]. In conclusion, berberine exerts beneficial effects on both metabolic and reproductive alterations associated with PCOS through multiple interconnected mechanisms. Experimental and clinical evidence suggests that berberine improves insulin sensitivity by modulating the PI3K/AKT signaling pathway and enhancing GLUT4 expression. In addition, berberine reduces inflammatory responses by decreasing the expression of mediators such as TNF-α, IL-6, NF-κB, and HAS2, while also exerting antiapoptotic effects through the regulation of Bax and Bcl-2 expression. These combined actions contribute to the restoration of ovarian morphology, improvement in follicular development, reduction in hyperandrogenism, and overall amelioration of endocrine and metabolic dysfunctions associated with PCOS (Figure 2).

In general, berberine is well-tolerated and has a favorable safety profile. Despite this, berberine is still classified as a dietary supplement. Its popularity among patients is due to its wide availability, the fact that it does not require a prescription, and its natural origin, factors that many people associate with safety and fewer side effects. However, given the current lack of sufficient scientific evidence regarding its safety and efficacy, it should not be routinely prescribed as a treatment for PCOS [104]. Treatment with berberine may be safe as it does not increase the overall incidence of adverse events or the risk of hypoglycemia [105]. However, in terms of safety, metformin has a well-established history following decades of clinical use. Its most common side effects are nausea and diarrhea, and in rare cases it can cause lactic acidosis, particularly in patients with renal impairment [108]. Based on this, metformin remains the most reliable and well-supported option for treating PCOS, while berberine is used as an additional adjunct treatment, and its use should be supervised by a healthcare professional.

### 5.2. Terpenoids

Terpenoids are organic compounds, also known as isoprenoids, because they are formed from single or multiple units of isoprene (2-methyl-1,3-butadiene), a five-carbon hydrocarbon. The molecular structure of terpenoids can be linear, cyclic, or a combination of both, and according to their number of isoprene units they can be classified as hemi- (C5), mono-(C10), sesqui- (C15), di-(C20), sester- (C25), tri-(C30), tetra-(C40), and polyterpenes (containing more than 8 isoprene units) [109,110,111,112,113].

They are present in fungi, animals and plants, the latter being those that present a great variety of terpenoids, where more than 80,000 compounds have been described, making them the largest class of metabolites of plant origin [111,114,115]. Terpenoids have been described as having hypoglycemic, anti-inflammatory, antioxidant, antibacterial, antifungal, antiangiogenic, and antimetastatic properties [116], and have therefore been used in the treatment of various pathologies [117,118,119,120], including PCOS, where they have been used as complementary therapy to address hormonal and metabolic alterations [120]. Table 2 shows the effects of terpenoid use in preclinical studies of animal models with PCOS induction.

The effects of some of the terpenoids that have been used in clinical studies for the treatment of PCOS are described below.

Astaxanthin is a tetraterpenoid belonging to the carotenoid group. It is a fat—soluble red pigment found in algae, yeast, salmonid species (such as salmon and rainbow trout), and some crustaceans (shrimp, krill). It is a potent antioxidant with anti-inflammatory, cardioprotective, and neuroprotective effects, as well as antitumor properties. Therefore, it has been used as an adjunct to the treatment of diabetes mellitus, dementia, Alzheimer’s disease, Parkinson’s disease, cancer, as well as liver disease and CVD [131,132,133,134].

Clinical studies have shown that in infertile women with PCOS, treatment with astaxanthin (12 mg/day, orally) for 60 days resulted in an increase in total antioxidant capacity (TAC) in follicular fluid, without changes in other oxidative stress markers such as MDA or SOD. Furthermore, it increased the quality of oocytes and embryos, possibly because of reduced oxidative stress [135]. Subsequently, the same treatment regimen for 8 weeks was shown to improve glucose metabolism, insulin sensitivity, and some lipid profile and oxidative stress markers, in addition to reducing fasting glucose and insulin levels [136]. These results led to the conclusion that astaxanthin can be used as an effective and safe supplement for the treatment of patients with PCOS.

In a group of infertile women with PCOS, who received 6 mg of astaxanthin for 8 weeks, the serum concentration of IL-6 and IL-1β decreased, and improvements were observed in some reproductive outcomes such as the number of oocytes retrieved, oocyte count, oocyte maturity rate, and the number of frozen embryos. Therefore, the authors concluded that pretreatment with astaxanthin, due to its anti-inflammatory effects, could improve the results of assisted reproduction techniques in these patients [137].

On the other hand, Fu et al. [138] showed that in PCOS patients who received astaxanthin supplementation for 3 months, AMH, fasting insulin, IR, basal LH concentration, and testosterone levels decreased. Furthermore, embryo quality and pregnancy outcomes improved [138].

Thymoquinone is a monoterpenoid and the main bioactive component of *Nigella sativa*, an herbaceous plant commonly known as black seed or black cumin. It is used as a spice in food and for medicinal purposes in the treatment of cancer, diabetes, obesity, and hypertension [139,140,141].

Naeimi et al. [142] evaluated the effect of *N. sativa* on oligoamenorrhea in women with PCOS. Treatment with *N. sativa* oil (2 capsules of 500 mg each) for 16 weeks resulted in a decrease in the menstrual interval (from 86 to 45 days) and an increase in menstrual cycle frequency, suggesting that it could be used as an alternative treatment for menstrual irregularities in patients with PCOS [142].

Coenzyme Q10 (CoQ10), also known as ubiquinone, is a polytepenoid naturally produced by the body, where it plays an important role in energy production and as an antioxidant. Foods rich in CoQ10 include fish, meat, nuts, and some oils. Fruits, vegetables, and grains also contain it, but in lower concentrations. CoQ10 has been considered a potential candidate for the treatment of various cardiovascular, neurodegenerative, and neuromuscular diseases, among others. Due to its beneficial effects, it is the third-most consumed nutritional supplement [143,144,145].

In women with PCOS who received 100 mg/day of CoQ10 for 12 weeks, hirsutism, serum testosterone, DHEAS, and MDA levels decreased, while serum SHBG and TAC levels increased. Furthermore, an improvement in the patients’ mental health was observed, with decreases in depression and anxiety scores [146].

Taghizadeh’s group [147] analyzed the effect of CoQ10 in overweight and obese women diagnosed with PCOS. Patients received 200 mg CoQ10 capsules daily for 8 weeks, and the study observed that this treatment decreased serum concentrations of markers of inflammation and endothelial dysfunction [147]. Furthermore, Izadi et al. [148] showed that CoQ10 supplementation for 8 weeks had beneficial effects on the metabolic and hormonal profile of women with PCOS, decreasing serum concentrations of fasting glucose, insulin, IR, and testosterone [148].

Izhar et al. [149] evaluated the effect of combined clomiphene citrate and CoQ10 therapy in women with PCOS, observing a higher proportion of women who ovulated and a higher conception rate than those who received only clomiphene citrate; therefore, it was concluded that CoQ10 is a suitable supplement to induce ovulation in women with PCOS who show resistance to clomiphene citrate [149].

Preclinical and clinical evidence demonstrates the therapeutic potential of terpenoids in the management of PCOS, due to their multiple beneficial effects as antiandrogenic, antidyslipidemic, antioxidant, and anti-inflammatory agents (Figure 2). And although their use as adjuncts or alternative therapies in the treatment of PCOS has been proposed, further studies are needed.

### 5.3. Phenolic Compounds

Phenolic compounds are molecules that are characterized by having one or more aromatic rings (benzene) with one or more groups OH and may have an additional distinctive functional group [150,151,152].

They are a group of secondary metabolites produced primarily by plants. They are found in cereals, fruits, vegetables, roots, olive oil, wine, tea, and other plant products. They have antimicrobial, antioxidant, and anti-inflammatory properties, so their regular intake is associated with a lower risk of developing certain types of cancer and cardiovascular and neurological diseases [151,152].

Phenolic compounds are divided into several subclasses according to their chemical structure, including (Figure 2): phenolic acids, flavonoids, tannins, stilbenes and lignans [150,151]. The phenolic compounds are used for the treatment of PCOS. It is worth noting that most polyphenols undergo extensive biotransformation mediated by the gut microbiota before being absorbed by the human body [153].

#### 5.3.1. Phenolic Acid

Vanillic acid is a phenolic acid found in various plants and fruits, including rice, thyme, cherries, oranges, green tea, wine, and beer. It is the oxidized form of vanillin (a phenolic aldehyde used as a flavoring agent in the food industry, which can be isolated from the pods of the orchid *Vanilla planifolia).* Vanillic acid has been described as having phytoestrogenic, antibacterial, cardioprotective, anti-inflammatory, antioxidant, hepatoprotective, and antitumor activity [154,155,156].

A preclinical study in rats with letrozole-induced PCOS showed that treatment with vanillic acid (25, 50, and 100 mg/kg orally) for 15 days had dose-dependent effects, with the 100 mg/kg dose showing the most pronounced effects. This dose resulted in reduced body and ovarian weight, decreased LH and testosterone levels, and reduced oxidative stress. Furthermore, ovarian histology revealed a decrease in the number of cysts and the presence of normal, healthy follicles at different developmental stages. These effects were similar to those observed with clomiphene citrate treatment (1 mg/kg orally). This evidence demonstrates the efficacy of vanillic acid in the treatment of PCOS [157].

#### 5.3.2. Flavonoids

Flavonoids are a subclass of polyphenols found in fruits, vegetables, tea leaves, nuts, flowers, and plants [158]. They are divided into several subcategories, including flavanols, flavonols, flavones, isoflavones, chalcones, and anthocyanidins [159]. Catechins are the most abundant flavanols in tea, while flavanones such as naringenin and paclitaxel are derived from citrus fruits. Flavones such as luteolin and apigenin are found in celery, tea, red bell peppers, and oranges [159]. Beans and their derivatives are the main sources of isoflavones, with genistein and glycitein being representative compounds. Quercetin is the most studied flavonol and is found in onions, kale, leeks, and other foods. Chalcone is a natural flavonoid that acts as a bioprecursor in flavonoid synthesis [160]. Anthocyanidins, such as anthocyanins, delphinidins, petunidins, peonidins, malvidins, and pelargonidins, are the natural pigments present in plants that give color to flowers and fruits [161,162].

Among its most studied properties, its antioxidant activity stands out, which helps prevent damage caused by the increase in free radicals originating from a pathological condition. This is achieved through the elimination of ROS, the activation of antioxidant enzymes, and the inhibition of various oxidases, such as xanthine oxidase (XO), cyclooxygenase (COX), lipoxygenase, and PI3-K [163]. Furthermore, flavonoids have anti-inflammatory effects, as they can inhibit the development of inflammation by preventing the production of inflammatory mediators such as prostaglandins and leukotrienes [164]. Flavonoids have also been shown to have antitumor activity [165]. In the context of PCOS, whose etiology is multifactorial due to the interaction of genetic, hormonal, inflammatory, and oxidative factors [143], flavonoids have emerged as therapeutic candidates [166,167,168,169]. This is because these compounds can improve insulin sensitivity by activating the AMPK pathway, decrease IR-associated signaling, reduce oxidative stress, and attenuate systemic inflammation [166].

Research on the therapeutic effects of flavonoids in PCOS has shown that these compounds can mitigate the reproductive and metabolic alterations associated with the condition. For example, quercetin has been reported to be involved in metabolic and endocrine processes related to PCOS development, as it acts as a PI3-K inhibitor. In a murine model of PCOS induced with testosterone propionate or letrozole, treatment with 30–150 mg/kg of quercetin exerts beneficial metabolic and endocrine effects, as this flavonoid significantly reduces serum concentrations of insulin, testosterone, and LH, and improves the lipid profile, resulting in improvements in the typical PCOS abnormalities. At the molecular level, quercetin has been shown to inhibit PI3-K activity and decrease the expression of the *CYP17A1* gene. These findings indicate that quercetin could attenuate the metabolic and endocrine alterations in PCOS, and it is postulated that the inhibition of PI3-K in theca cells results in decreased androgen production by decreasing the expression of the *CYP17A1* gene [170,171].

Another study observed that an animal model of PCOS induced with DHEA and treated with 25 mg/kg of quercetin or metformin orally resulted in lower concentrations of serum free testosterone, LH, and the LH/FSH ratio. Furthermore, the number of atretic follicles and ovarian cyst formation were reduced in rats with PCOS. The authors propose that quercetin is as effective as metformin in reducing hyperandrogenism and improving the function of HPA axis, suggesting that both substances can restore ovarian folliculogenesis and decrease atresia by reducing hyperandrogenism in rats with PCOS [172].

In addition to quercetin, other flavonols such as myricetin have been studied as potential therapies for PCOS. In mouse models of PCOS, brown adipose tissue (a tissue capable of eliminating and oxidizing glucose and lipids in organisms, which has been of interest in combating obesity and its associated metabolic diseases) was stimulated through the administration of myricetin. In this study, Zheng et al. [173] showed that a βweek treatment with myricetin improved metabolic capacity and insulin sensitivity by activating brown adipose tissue in mice with PCOS. Furthermore, an increase in the number of corpora lutea and a decrease in ovarian cyst formation were observed. Additionally, LH concentrations and the estrous cycle were restored. The above suggested that myricetin treatment dramatically improves ovarian dysfunction and metabolic alterations in animals with PCOS and offers a new perspective for the treatment of the pathology [173].

Although there is limited information on the role of myricetin in the development and persistence of PCOS, some authors, such as Aswal et al. [174], suggest that this flavonol could be a candidate for PCOS treatment. This is supported by recent studies demonstrating that myricetin in animals with PCOS has an affinity for PI3-K and NF- κB (−8.6 and −7.0 kcal/mol, respectively), indicating its potential to improve insulin sensitivity. Furthermore, the therapeutic efficacy of myricetin was evaluated in rats with a high-fat diet and PCOS. Treatment with myricetin at varying doses significantly reversed PCOS associated with metabolic and reproductive abnormalities. This is since a dose of 300 mg/kg of myricetin showed substantial improvements in body weight, as well as in breast milk volume (BMV) and the estrous cycle; it restored hormonal balance and improved IR, β-cell function, serum lipid profile, oxidative stress, and histology in ovarian tissues. The favorable results observed in rodents treated with myricetin suggest that this flavonol is safe and effective for the treatment of PCOS [174].

It is also known that PCOS is associated with increased oxidative stress, characterized by high concentrations of ROS and markers of cellular damage, which contributes to the development of IR, chronic inflammation, and ovarian dysfunction, exacerbating the metabolic and reproductive complications of PCOS [175]. In a study conducted in rats with IV-induced PCOS, the effect of a hydroalcoholic extract of green tea, rich in antioxidant catechins (flavanols), was evaluated. After treatment with different doses of catechins (50, 100, and 200 mg/kg), LH concentration, body weight, ovarian weight, and IR decreased significantly. Furthermore, ovarian morphology showed a greater number of follicles and corpora lutea, and a lower number of cystic follicles in the ovary. In addition, the follicles showed a reduction in the thickness of the theca cell layers, possibly mediated by increased lipolysis and decreased hypertrophy of this layer, resulting in decreased androgen levels. These results suggest that green tea may be useful in the treatment of PCOS and associated metabolic and reproductive disorders [176].

Genistein, an isoflavone found in various legumes such as beans, chickpeas, peas, and in higher concentrations in soybeans, has been shown to act as a phytoestrogen due to its ability to mimic estrogens and decrease circulating androgens [177,178]. This compound has demonstrated protective effects against metabolic and hormonal disorders, both experimentally and in women with PCOS.

In rats with estradiol valerate (EV)-induced PCOS, marked degeneration was observed in the granulosa and theca cell layers that make up developing follicles. These alterations are associated with the absence of corpora lutea, indicative of anovulation. In another study in rats with the same condition treated with 1 mg/kg BW of genistein for 14 days, LH concentrations decreased and FSH concentrations increased. This effect was attributed to the structural similarity of genistein to 17β estradiol, which promotes GnRH release. Furthermore, administration of the phytoestrogen significantly decreased glucose and insulin concentrations, as well as IR, which in turn contributed to reducing excess testosterone. Healthy follicles and corpora lutea were also observed in these animals. With these findings, the authors suggest that genistein exerts beneficial effects in PCOS rats by modulating gonadotropin and androgen synthesis, controlling IR, and improving follicular development [179].

Rajaei’s group in 2019 showed that in animals with EV-induced PCOS, there was an increase in oxidative stress, a decrease in antioxidant activity, cyst formation, and alteration of follicular structure. However, in those PCOS rats that were administered 1 mg/kg of genistein subcutaneously for 14 days, the follicular quality was similar to that of the group without the pathology, MDA levels in plasma and ovaries decreased significantly (*p* ≤ 0.0001), as did TAC (*p* ≤ 0.0001). This evidence led the authors to conclude that oxidative stress and decreased antioxidant activity are important characteristics of PCOS and that treatment with genistein can reduce these harmful effects [180].

In another study conducted in Sprague-Dawley rats with letrozole-induced PCOS, the effects of genistein on IR, inflammation, oxidative stress, lipid profile, and ovarian histopathological indices were evaluated. Following administration of saline, metformin (150 mg/kg BW), or genistein (20 mg/kg BW) for 42 days, genistein was found to significantly reduce fasting insulin concentrations and the HOMA-IR, as well as serum MDA and TNF-α concentrations, and to increase SOD activity. Histopathological analyses showed increased luteinization and a reduction in the number of cysts. Taken together, these findings indicate that genistein administration produced a significant decrease in oxidative, inflammatory, glycemic, and histopathological alterations associated with PCOS, supporting its potential as a therapeutic alternative [181].

In 25-day-old mice with DHEA-induced PCOS, intraperitoneal treatment with genistein (8.75 mg/kg for 15 days) restored the estrous cycle, reduced ovarian cysts, normalized testosterone, LH, and AMH levels, and reduced the oxidative stress biomarkers SOD and catalase (CAT). Additionally, in granulosa cell cultures from these patients treated with genistein, they observed the restoration of ATP levels and membrane potential, as well as increased expression and activity of nuclear factor erythroid 2-related factor 2 (Nrf2) and Foxo1 (regulators of cellular antioxidant defense). Based on these results, they propose that genistein has therapeutic potential generated through its binding to the estrogen receptor, thereby increasing antioxidant defense via ER–Nrf2–Foxo1–ROS [182].

Another study evaluated the efficacy of daidzein and genistein, present in soy tempeh extract. The percentage yield of the extract obtained was 1.87%, and daidzein was found to be the predominant compound, representing 73.34% of the total isoflavones. This compound, administered at a dose of 250 mg/kg BW, was used in Wistar rats in which PCOS was induced with letrozole. Daidzein restored estrous cycles more effectively than metformin. Furthermore, testosterone concentrations decreased. The research findings suggested that tempeh extract acts as an antiandrogen, and therefore, it was postulated that this extract could be used as a dietary supplement for the management of PCOS [183].

In a murine model of letrozole-induced PCOS, daidzein, along with genistein, played a central role in hormonal restoration and improved ovarian function; however, its low solubility and bioavailability limit its efficacy. Research has shown that the probiotic *Bacillus Coagulase* significantly enhances the bioactivity of these isoflavones due to the action of its enzyme CGTase, which can increase their solubility through transglycosylation. The combined treatment of soy isoflavones (100 mg/kg BW) with *Bacillus coagulans* resulted in the recovery of the estrous cycle, reduction in oxidative stress, normalization of the lipid and glycemic profile, and restoration of ovarian physiology. Taken together, the findings highlight that daidzein in the presence of the probiotic is a key component in the attenuation of PCOS symptoms [184].

The effects of genistein have also been evaluated in women with PCOS. In a prospective pilot study lasting six months, twelve Caucasian women with PCOS received 36 mg/day of genistein. Phytoestrogen supplementation improved TC levels. This decrease was primarily due to a lower serum concentration of LDL cholesterol. Additionally, plasma HDL cholesterol concentrations increased slightly during treatment, although not significantly. However, the combination of these changes in lipoprotein concentrations led to a significant decrease in the LDL-HDL ratio. No significant effects were detected on anthropometric or hormonal parameters, menstrual cyclicity, or glucose–insulin metabolism. These results suggest that the therapeutic benefits of genistein in PCOS are mainly limited to improving the lipid profile [185].

In another quasi-randomized study involving 146 patients with PCOS, participants were assigned to different experimental groups. The first group received 18 mg of genistein twice daily, while the second group, a control group, received a placebo for 3 months. Hormonal and lipid profiles were assessed before and after treatment. Following the intervention, no significant changes were observed in HDL or FSH levels; however, the group receiving genistein showed significant decreases in LH, TG, LDL, DHEAS, and testosterone. These findings suggest that genistein supplementation could contribute to the prevention of cardiovascular and metabolic disorders in women with PCOS by improving their hormonal and lipid profiles [186].

In women with PCOS, anthocyanins improve IR and reduce inflammatory processes. Furthermore, studies in rodents have further clarified the role of these flavonoids in PCOS. In a study by Moshfegh et al. [187], mice with a testosterone enanthate-induced PCOS-like phenotype were used to evaluate the effect of saffron petal extract (SPE) and saffron petal anthocyanins (SPA). Both compounds were shown to decrease previously elevated concentrations of LH, testosterone, and estrogen. Furthermore, both SPE and SPA normalized the expression of genes related to gonadotropin receptors (*FSHR, LHR*), steroid receptors (*Pgr*, *Esr1*), as well as inflammatory markers and mediators such as TNF*-α*, IL*-1β*, IL *-6,* interleukin 18 *(IL-18),* NF*-κB*, NF*-κB p65,* and nuclear factor kappa *(IκB*). They also normalized the expression of antioxidant enzymes such as GPx, SOD, CAT, glutathione S-transferase (GST), and glutathione (GSH). Additionally, they reversed the androgenic environment in reproductive tissues toward an estrogenic state. Taken together, the results suggested that both compounds attenuate PCOS symptoms by modulating steroidogenesis, inflammation, and oxidative stress [187].

There are several types of anthocyanins, such as cyanidin-3-O-glucoside (C3G), malvidin-3-(6-caffeoyl)-glucoside, and delphinidin-3-O-glucoside, about which little research has been performed to date on their role in the development and persistence of PCOS. For example, an article published in 2023 mentioned that malvidin-3-(6-caffeoyl)-glucoside is found in the medicinal plant *Centratherum anthelminticum* (CA), which has recognized antioxidant properties and Antihyperlipidemic effects. In the study by Shoaib et al. [188], the effect of cacti seed extract was evaluated in Wistar rats with PCOS induced by EV and a high-fat diet. Treatment with cacti (250–750 mg/kg/day) for 28 days reduced cholesterol, TG, IR, and inflammatory markers (IL-6, NF-κB), and improved oxidative balance (SOD, CAT, GSH, MDA). It also normalized reproductive hormone parameters (such as increased progesterone and FSH concentrations and decreased LH) and restored follicular development, demonstrating the recovery of the estrous cycle. Taken together, these findings demonstrated that cacti exert anti-PCOS effects attributable to its antidiabetic, anti-inflammatory, antihyperlipidemic, and antioxidant actions [189].

Later, Behinska and colleagues in 2025 [188], mentioned that C3G, a dietary flavonoid from the anthocyanin group, protects ovarian cells from oxidative stress, while inhibiting tumor growth and inducing apoptosis in ovarian and cervical cancer cells. However, some studies indicate that C3G could mitigate reproductive disorders such as PCOS, although its low bioavailability and the need for improved delivery methods still present challenges [188].

Based on the evidence, it is proposed that flavonoids could be an effective therapeutic alternative for addressing the metabolic and reproductive alterations caused by PCOS. Basic experimental and clinical evidence suggests that flavonoids, through the activation of different cellular mechanisms, restore ovarian morphology, decrease androgen concentration, improve insulin sensitivity, reduce inflammation, and restore ovarian cyclicity in PCOS. However, clinical studies are still needed to confirm their efficacy in women. In summary, natural compounds such as flavonoids could complement conventional PCOS therapies and open new avenues for integrative treatment (Figure 2).

### 5.4. Essential Fatty Acids (Omega-3 and Omega-6)

Linoleic acid (omega-6) and α -linolenic acid (omega-3) are considered essential fatty acids because the human body cannot biosynthesize them, making it necessary to obtain them through diet. Arachidonic acid is formed from linoleic acid, and eicosapentaenoic acid (EPA) and docosahexaenoic acid (DHA) are formed from α- linolenic acid; these fatty acids depend on their precursors to maintain homeostasis [190].

Humans can synthesize only a limited amount of EPA and DHA from α-linolenic acid (approximately 5%); therefore, these fatty acids must be obtained from the diet [191]. The recommended intake of omega-3 fatty acids varies according to life stage, sex, and even physiological state, and can range from 0.5 to 1.6 g per day. Sources of fatty acids include algae, shellfish, fish, vegetables such as purslane and spinach, nuts, and some types of oils [192,193].

Essential fatty acids are structural components of cell membrane phospholipids, which confer fluidity, flexibility, and selective permeability that depend on the amount of fatty acids they contain [194,195]. It has been observed that a higher proportion of polyunsaturated fatty acids relative to saturated fatty acids increases cell membrane fluidity [190]. Arachidonic acid, EPA, and DHA serve as substrates for the formation of eicosanoid lipid derivatives (derivatives of arachidonic acid and EPA) and docosanoids (derivatives of DHA), which participate in regulating cellular metabolism [196].

Fatty acids participate in the regulation of the immune, digestive, nervous, cardiovascular, reproductive, and endocrine systems [191,195,197]. Arachidonic acid, as a component of cell membranes, is released by activation of phospholipase A2 during the early stages of an inflammatory process. It is then metabolized by lipoxygenase and COX enzymes into bioactive eicosanoids, including prostaglandins (molecules that promote inflammation, pain, fever, and increased vascular permeability), leukotrienes (such as B4, C4, and D4, potent pro-inflammatory agents that increase vascular permeability and stimulate the release of inflammatory cytokines), and thromboxanes (mediators of the local immune response). Diets rich in EPA and DHA increase the proportion of these fatty acids in cell membranes, which, through competition, reduces the content of arachidonic acid and decreases the production of pro-inflammatory products derived from omega-6 fatty acids [198]. A diet high in omega-3 and low in omega-6 is associated with a decrease in inflammatory molecules such as thromboxane II, prostaglandins, IL-1 and IL-8, and leukotrienes [190]. Other relevant effects of omega-3 fatty acid consumption include its effectiveness in improving insulin sensitivity and reducing TG [190]. The incorporation of omega-3 derivatives from the diet increases the proportion of these fatty acids in cell membranes, which, through a competitive effect, reduces arachidonic acid content and increases membrane fluidity. These changes may improve the responsiveness of membrane-embedded proteins, including the insulin receptor, thereby enhancing cellular sensitivity to external stimuli [193].

The incorporation of essential fatty acids into the diet has been included as part of PCOS treatments, due to their favorable effects on obesity, inflammation and IR [199,200,201]. This has been shown in animal models in which PCOS is induced, as well as in women with the condition, as explained below.

In female rats that had PCOS induced by a daily release implant of 5 *α*-dihydrotestosterone, Ma’s group [202] showed that treatment with food containing omega-3:omega-6 fatty acids, in a 1:15 ratio, for 30 days, increased ovarian weight, FSH and estradiol concentration and decreased testosterone concentration, compared to rats treated with fatty acids in a 1:3 ratio. In rats treated with a 1:15 ratio of fatty acids, an increase in the expression of steroidogenic enzymes such as sterol 14α- demethylase (CYP51), aromatase (CYP19), steroidogenic acute regulatory protein (StAR), and 3β-HSD was observed when compared to those treated with a 1:3 ratio. This allowed the authors to suggest that supplementation with omega-3 and omega-6 fatty acids in a 1:15 ratio improves the endocrine alterations in PCOS [202].

In 2020, Wang’s group [203] showed that in rats with letrozole-induced PCOS, daily treatment for 8 weeks with 1 mL/kg of linseed oil, administered via gastric tube, restored estrous cycles and ovulatory capacity, increased FSH, progesterone, and estradiol concentrations, and decreased the LH/FSH ratio and testosterone levels. Regarding the metabolic profile, linseed oil treatment reduced body weight, dyslipidemia, and IR. As for cytokines involved in inflammatory processes, linseed oil treatment improved plasma and ovarian concentrations of the interleukins IL-1β, IL-6, IL-10, IL-17, and TNF- α, as well as MCP-1 and serum lipopolysaccharide concentrations. The authors conclude that flaxseed oil included in the diet improved PCOS alterations through the regulation of the steroid hormone-inflammation axis, which may contribute to understanding the pathogenesis of the syndrome and serve as a cost-effective treatment in the management of PCOS [203].

The beneficial effects of fatty acids on the regulation of the hormonal and metabolic profile have also been shown in the rat with PCOS induced by the injection of EV, by treatment for 16 weeks with different presentations of fatty acids: (1) synthetic omega-3 (contains α -linolenic acid, EPA and DHA), (2) flaxseed oil (contains α -linolenic acid) or (3) fish oil (contains EPA and DHA) [204].

Furthermore, fatty acid supplementation has shown favorable effects in clinical studies of women with PCOS. The group of Oner and Muderris [205] showed that in women with PCOS without obesity, a 6-month oral treatment with 1500 mg of omega-3 fatty acids resulted in a decrease in BMI, hirsutism, insulin concentration, and HOMA-IR, without changes in glucose concentration during the treatment period. Regarding the hormonal profile, a decrease in testosterone and LH concentrations and an increase in SHBG concentration were observed. TNF-α and resistin, molecules associated with insulin sensitivity, increased after treatment. These findings led the authors to suggest a positive correlation between insulin sensitivity, hirsutism, and BMI with omega-3 use, and that treatment with these fatty acids has the advantage of being less cost-effective for PCOS [205].

Nasri et al. [206] showed that in women with PCOS, supplementation for 12 weeks, twice daily, with 1000 mg of omega-3 fatty acids (from flaxseed oil) combined with 400 mg of α -linolenic acid (omega-6) increased the expression of PPAR-γ mRNA involved in the insulin signaling pathway, adipocyte differentiation, fatty acid storage, and glucose metabolism [206]. However, the authors mention that the mechanisms through which omega-3 fatty acids regulate PPAR-γ signaling remain largely unknown. The study also showed a decrease in the expression of the mRNA of the oxidized LDL receptor (LDLR) in peripheral blood mononuclear cells of women with PCOS; however, the authors note that the role of fatty acids in the LDLR regulatory pathways is still unknown in the literature, since there are data showing that omega-3 supplementation can increase or decrease LDLR values, which seems to depend on the source of the fatty acid, the concentration, the characteristics of the participants in the different studies, as well as the duration of the study [207,208].

Najdgholami’s group [209] showed that in a group of 35 women with PCOS, supplementation with 30 g of ground flaxseed (rich in omega-3 fatty acids), along with lifestyle modifications, for 12 weeks, increased FSH concentration and decreased the LH/FSH ratio. However, no changes were observed in the concentrations of AMH, LH, estradiol, androstenedione, or DHEAS when compared to women with the syndrome who did not receive flaxseed treatment. The authors suggested that flaxseed supplementation has a potential effect on normalizing gonadotropin secretion and should be considered complementary to the management of patients with the syndrome [209].

Based on the above results, we can conclude that, in general, the role of fatty acids in regulating the alterations in women with PCOS is favorable (Figure 2).

### 5.5. Inositol

Inositol was first identified in animal muscle tissue by J.J. Scherer in 1850 [210], and it occurs naturally in various foods, including nuts (almonds, walnuts, and Brazil nuts), cereals (oats and bran), legumes (beans and peas), and fruits (melon and citrus fruits, except lemons) [211]. It is a physiological compound belonging to the sugar family [212]. Nine stereoisomers derived from the epimerization of hydroxyl groups are known, among which myo-inositol (MI) and D- chiro-inositol (DCI) are the most abundant in the human body and of the greatest therapeutic interest [212,213,214], in addition to being the two main stereoisomers of inositol present naturally in animal and plant cells, either in their free form or as components bound to phospholipids or inositol phosphate derivatives [210].

The MI and DCI play key roles as intracellular second messengers in insulin signaling. MI is biotransformed into myo- inositol phosphateglycan (MI-IPG), which participates in cellular glucose uptake, while DCI gives rise to D-chiroinositol phosphateglycan (DCI-IPG), involved in glycogen synthesis [215]. Furthermore, MI-IPG enhances glucose uptake in the ovary and is essential for FSH signaling, while DCI-IPG is involved in insulin-mediated androgen production. Both isomers can reduce LH and testosterone levels, as well as the LH/FSH ratio, counteracting hyperandrogenism and its clinical manifestations, such as hirsutism and acne [214].

MI is also part of membrane phosphatidylinositols, precursors of second messengers such as inositol triphosphate (IP3) and diacylglycerol (DAG), which regulate various hormonal activities, including the action of insulin, FSH and thyroid-stimulating hormone (TSH) [210,216].

The therapeutic potential of MI and DCI has been demonstrated in various experimental models. In rats with letrozole-induced PCOS, administration of MI at a dose of 400 mg/kg/day for 15 days reduced glucose, improved the lipid profile (decreased TG by 30% and increased HDLC by 25%), and induced the AMPK/GLUT4 pathway, which promotes insulin sensitivity and lipid metabolism [217].

Regarding inositol therapy for women with PCOS, there is a general consensus that their profile requires a careful and comprehensive preliminary analysis of the patient, considering, among other aspects, their reproductive expectations since pregnancies were reported in women with the pathology who received MI in combination with folic acid [215].

In a 2017 study by Fruzzetti’s group [213] in women with PCOS with IR and/or hyperinsulinemia, metformin (1500 mg/day) and methylprednisolone (MPI) (4 g/day) were compared over 6 months. Both treatments improved insulin sensitivity, significantly reduced IR, and normalized the menstrual cycle in approximately 50% of the participants. The authors concluded that both metformin and MPI are equally effective in improving the parameters in patients with PCOS [213].

Several studies have been conducted to identify the optimal dosage for patients with this condition. In a study by Nordio et al. [214] analyzing the effect of a combination of MIand injectable nephrazole in a 40:1 ratio (2 g), twice daily (morning and evening) for three months, participants experienced ovulation and increased levels of FSH, SHBG and 17β-estradiol, along with decreased levels of LH, free testosterone, HOMA-IR, and basal and postprandial insulin concentrations [214]. Subsequently, in 2022, Kachhawa et al. evaluated women with PCOS treated for six months with a combination of MI and nephrazole (550 mg + 150 mg) in a 3.6:1 ratio twice daily, also observing menstrual cycle regulation and improved IR. The results of these studies show that even at low doses of inositol, there is improvement in both reproductive and metabolic parameters in women with PCOS [218].

Several recent studies have compared the efficacy of inositol combinations versus metformin in women with PCOS. When 1951 mg of MI + 49 mg of nephrazole are administered daily in a 40:1 ratio for 12 weeks to these patients, the same efficiency in improving ovarian function by promoting ovulation is achieved, and the potential side effects are avoided as with the daily oral dose of 2000 mg of metformin, which is the standard treatment for women with PCOS [219].

Regarding the MI/DCI ratio, it is crucial to note that while it remains the formulation with the strongest scientific support, the majority of the studies establishing this ratio are based on specific cohorts of Caucasian women with PCOS [214,219,220,221]. Previous studies indicate that this condition exhibits significant metabolic and clinical heterogeneity among different ethnic groups [222,223]. For example, due to ethnic variations in metabolic risk phenotypes [224], populations with a higher genetic predisposition to metabolic syndrome are those from Asia and the Americas, while in European women this risk is moderate [225]. Since a specific therapeutic ratio for different ethnicities has not yet been established, it is clear that multi-ethnic clinical trials are needed to determine whether personalized MI/DCI ratios are required based on each patient’s genetic and metabolic background.

Other treatment alternatives for PCOS have also been studied involving the combination of inositols plus prebiotics, since evidence in the literature showed that adding a prebiotic molecule, such as α-lactalbumin (α-LA), in a formulation with inositol, improves its intestinal absorption [226] and consequently its clinical efficacy [227,228], a fact that was corroborated by Ozay’s group in 2025, who treated 30 adult patients for 12 to 16 weeks divided into two groups: one received twice a day 550 mg of MI, 13.8 mg nephrazole, 14.1 mg α-LA and 200 µg of folic acid; the other group received 500 mg of metformin also twice a day. Both treatments improved the metabolic profile (insulin, HOMA-IR and fasting glucose) and reduced androstenedione concentrations; however, only the group treated with inositols plus prebiotics showed a significant decrease in free testosterone concentrations [221].

It should be noted that, according to the 2023 International Evidence-Based Guidelines for the Assessment and Management of PCOS, inositol exhibits potential benefits in improving metabolic parameters, although clinical evidence remains limited regarding aspects such as ovulation rates and the reduction in hyperandrogenism. Currently, its use for fertility in women with PCOS is considered an experimental therapy, and the guidelines emphasize that there is still insufficient evidence to recommend specific types, doses, or combinations, underscoring the need for further standardized clinical trials [1].

Based on this evidence and the fact that no adverse or side effects have been reported to date associated with treatment with inositol or its derivatives, these compounds represent a promising therapeutic adjunct for patients with PCOS. Their potential to improve metabolic and reproductive profile (Figure 2) is well recognized; however, their clinical application remains experimental. It is essential to inform patients about both the potential benefits and risks of their use, and to promote further high-quality research on these molecules.

## 6. Conclusions

PCOS is a complex and heterogeneous endocrine–metabolic disorder that requires a comprehensive and personalized therapeutic approach. According to the 2023 International Evidence-based Guideline for the Assessment and Management of PCOS, lifestyle interventions including diet, physical activity, and behavioral strategies remain the first-line treatment for all women with PCOS. Although current pharmacological treatments such as metformin, oral contraceptives, and ovulation induction therapies are effective for managing specific clinical manifestations, they do not completely address the multifactorial pathophysiological mechanisms underlying the syndrome.

Current evidence indicates that natural bioactive compounds may complement conventional therapeutic approaches through the modulation of interconnected molecular pathways involved in IR, oxidative stress, chronic inflammation, mitochondrial dysfunction, ovarian steroidogenesis, and follicular apoptosis. These effects are associated with signaling pathways including PI3K/Akt, AMPK, NF-κB, MAPK, and CYP17A1 related steroidogenic regulation. Among the evaluated compounds, inositols currently exhibit the strongest clinical evidence supporting improvements in insulin sensitivity, ovulatory function, and metabolic outcomes in women with PCOS. Berberine and omega-3 fatty acids also demonstrate promising translational potential, whereas several polyphenols, terpenoids, and alkaloids show substantial mechanistic and preclinical promise but still require further clinical validation.

Despite these encouraging findings, the heterogeneity of study designs, formulations, dosages, and evaluated outcomes continues to limit direct comparisons and clinical standardization. Future research should prioritize phenotype-stratified clinical trials, standardized therapeutic endpoints, mechanistic biomarker analyses, long-term safety evaluations, and improved characterization of bioavailability and pharmacokinetic profiles. Integrating molecular, metabolic, and reproductive outcomes will be essential to determine the clinical applicability of bioactive compounds and their potential incorporation into evidence-based and personalized PCOS management strategies.

## Figures and Tables

**Table 1:** Biological effects of the use of berberine in preclinical models of PCOS.

Study Model	Treatment	Primary Endpoints	Secondary/Exploratory Outcomes	Biological Relevance	References
			BERBERINE		
Rats Letrozole-induced PCOS	100, 200 or 400 mg/kg/day for 28 days	**↓** HOMA-IR, ISI. **↑** ISI, the number of granule cells layers, follicles with oocytes.	**↓** MAPK. **↑** GLUT4, proteins IRS, PI3K, AKT.	Reduce IR values through a mechanism linked to GLUT4 upregulation via PI3K/AKT activation and MAPK pathway suppression, and partial restoration of normal tissue morphology.	Zhang et al. [93]
Rats Letrozole-induced PCOS	95 or 190 mg/kg/day for 28 days *	↓ T, E2, LH, HOMA-IR, FBG, FINS, number of apoptotic cells. ↑ P4, follicles in different developmental stages, CL, number of GC. = FSH	------	Alleviated the serum hormone imbalance and improved IR and exerts its proliferative and anti-apoptotic effects on ovarian granulosa cells, all in a PI3K/AKT dependent manner.	Yu et al. [86]
Rats Letrozole-induced PCOS	4.5 mg/kg/day for 28 days	**↓** T, LH, LH/FSH ratio, TC, TG, LDL-c, ghrelin, cystic dilated follicles. ↑ E2, FSH, HDL-c, CCK, PYY, GLP-1.	↑ PI3K, AKT proteins; mRNA levels of PI3K, GLUT4 and AKT.	The therapeutic effect of BBR+ 12.2 g/kg/day JPYSHZF improves hormone levels, ovarian function, glucose and lipid metabolism, and intestinal flora, thereby contributing to the treatment of obese PCOS rats. Also, can regulate IR by activating the PI3K/AKT signaling pathway	Zhang et al. [94]
Rats DHEA-induced PCOS	150 mg/kg/day for 42 days	**↓** T, HOMA-IR.	**↓** apoptosis regulating key signaling molecules (TLR4, LYN, PI3K, AKT, NF-kB, TNF-a, IL-1, IL-6, caspase-3) in the ovarian tissue.	It might have a protective effect on IR, inhibited cell apoptosis and reduced the inflammatory response caused by PCOS.	Shen et al. [95]
Rats DHEA-induced PCOS	40.5, 81 or 162 mg/kg/day for 28 days **	**↓** T, LH, FSH, HOMA-IR; FBG, FINS, TC, TG, OGTT (concentration-dependently), LDL, number of cyst follicles. ↑ HDL, recover estrous cycle.	------	It can protect normal histological structures of ovaries and plays a protective role in PCOS-IR rats, increasing insulin sensitivity, improving hyperandrogens and recovering abnormal blood lipids.	Li et al. [96]
Mice DHEA-induced PCOS	20 mg/100 g/day for 7 days	**↓** T, LH/FSH ratio, number of cyst follicles. **↑** number of CL, thickness of the GC layer. = LH, restoration of estrus cycle.	**↓** mRNA (in ovary) and protein (in serum) expression levels of MCP-1, IL-1β, and IL-6, HAS2 in ovary.	Attenuates inflammation in PCOS by reducing HAS2 expression suppressed inflammatory cytokine and restoration of the ovarian structure.	He et al. [97]
Mice DHEA-induced PCOS	250, 500, 1000 mg/kg/day for 14 days	**↓** T, LH, body weight (dose-dependent manner). **↑** number of CL, number of follicles at each stage.	**↓** IL-6, TNF-α.	It lowers the weight of PCOS mice, mitigates hyperandrogenemia and inflammatory state, and enhances recovery of ovulation	Lu and Lin [98]
Rats TP-induced PCOS	100 or 200 mg/kg/day for 56 days ***	↓ E2, LH, TC, OGTT, number of cysts. ↑ number of CL, endometrial thickness. = T, HOMA-IR, TG, INS.	↓ integrin αvβ3, protein expression of LPAR3. ↑ mRNA in granulosa cells and protein expression in ovary of LHCGR, CYP19A1.	BBR (dose-dependent manner) could improve ovulation in PCOS and the mechanism might be associated with up-regulating LHCGR and CYP19A1 and could also improve endometrial receptivity through down-regulating integrin αvβ3 and LPAR3.	Wang et al. [84]

AKT, AKT serine; BBR, berberine; CCK, cholecystokinin; CL, corpora lutea; CYP19A1, aromatase; DHEA, dehydroepiandrosterone; E2, 17β-estradiol; FBG: the fasting blood glucosa; FINS, Fasting insulin; FSH, follicle-stimulating hormone; GC, granulosa cells; GLUT4, glucose transporter 4; GLP-1, incretin hormone; HAS2, hyaluronan synthase 2; HDL, high density lipoprotein; HDL-c, High density lipoprotein colesterol; HOMA-IR, homeostatic model assessment-insulin resistance; IL-1, interleukin 1; IL-1β, interleukin β; IL-6, interleukin 6; INS, insulin; IR, insulin resistance; ISI, insulin sensitivity index; JPYSHZF, Jianpi Yishen Huazhuo formulation; LDL, low density lipoprotein; LDL-c, low density lipoprotein colesterol; LH, luteinizing hormone; LHCGR, Luteinizing hormone/choriogonadotropin receptor; LPAR3, lysophosphatidic acid receptor 3; LYN, LYN proto-oncogene; MAPK, Protein-Associated Kinase; MCP-1, monocyte chemotactic protein-1; OGTT, oral glucose tolerance test; P4, progesterone; PI3K, phosphatidylinositol 3-kinase; PYY, peptide YY; T, testosterone; TC, total colesterol; TG, total triglyceride; TLR4, protein levels of Toll-like receptor 4; TNF-α, tumor necrosis factor-α; TP, Testosterone propionate. * Metformin hydrochloride (50 mg/kg/day, gavage), an insulin sensitizing drug, has similar effects to BBR treatment [86]. ** Metformin (45 mg/kg/day, gavage), an insulin sensitizing drug, has similar effects to BBR treatment [96]. *** Metformin (183 mg/kg/day, gavage), an insulin sensitizing drug, has similar effects to BBR treatment [84]. Downward arrows indicate decreases, and upward arrows indicate increases.

**Table 2:** Biological effects of the use of terpenoids in preclinical models of PCOS.

Chemical Class/Compounds	Study Model	Treatment	Primary Endpoints	Secondary/Exploratory Outcomes	Biological Relevance	References
**MONOTERPENOIDS**						
Thymoquinone	Rats EV-induced PCOS	8 or 16 mg/kg for 30 days, IP.	**↓** glucose, TG, TC, LDL, LH, number of cysts. ↑ HDL, FSH, number of primary, antral, Graafian and CL follicles.	------	It improves ovarian morphology, ovulatory function, and metabolic disorders.	Taghvaee Javanshir et al. [121]
Thymoquinone	Rats Letrozole-induced PCOS	5 or 10 mg/kg. It was administered every 3 days for 30 days.	**↓** LH, T, LH/FSH ratio, atretic follicles, cysts, and ovarian weight. ↑ FSH, number of uni- and multilaminar, antral and Graafian follicles.	**↓** Bax expression and Bax/Bcl2 ratio.	It improves folliculogenesis, normalizes hormone concentration, and has an apoptotic and anti-atretogenic effect.	Alaee et al. [122]
Thymoquinone	Mice DHEA-induced PCOS	Oocyte incubation with 0, 1, 10 or 100 μM	↑ maturation, fertilization and blastulation rates.	**↓** Bax expression. ↑ Bcl2 expression.	It improves the developmental capacity of oocytes.	Eini et al. [123]
**TRITERPENOIDS**						
Astragaloside IV	Rats DHEA-induced PCOS	20, 40 or 80 mg/kg/day for 20 days. Subcutaneously injected	**↓** LH, FSH, T, insulin and glucose. ↑ number of rats with normal estrous cycles. It improves insulin resistance and follicular dynamics.	------	It normalizes hormone concentrations, the estrous cycle, follicular dynamics, and increases insulin sensitivity.	Wen et al. [124]
*β*-sitosterol	Mice DHEA-induced PCOS	25 mg/kg/day for 14 days. Intragastric treatment	**↓** LH, T. **↑** FSH, P4, endometrial thickness and markers of endometrial receptivity.	------	It normalizes hormone concentration, improves uterine condition, and promotes endometrial receptivity.	Yu et al. [125]
Lupeol	Mice DHEA-induced PCOS	40 mg/kg/day for 28 days, orally *	**↓** DHEA, T and atretic follicles. **↑** CL number.	**↓** MDA, TNF-α and mRNA of AR. **↑** TAC.	It has antiandrogenic, antioxidant, and anti-inflammatory effects.	Malekinejad et al. [117]
Lupeol	Mice DHEA-induced PCOS	40 mg/kg/day for 20 days, IP **	**↓** LH, T and atretic follicles. **↑** healthy follicles, CL and fertility rate.	**↓** MDA. **↑** TAC.	It reduces androgen concentration, allows the recovery of antioxidant capacity, and improves ovarian function and fertility.	Rezaei-Golmisheh et al. [126]
**TETRATERPENOIDS**						
Astaxanthin	Rats Letrozole-induced PCOS	10, 20 or 40 mg/kg/day for 7 days	Ovarian cysts persist.	**↓** MDA, TNF-α, IL-6 and NF-κB in ovary. **↑** SOD in ovary.	It reduces oxidative stress and inflammatory mediators in the ovary.	Toktay et al. [127]
Astaxanthin	Rats Letrozole-induced PCOS	10, 20 or 40 mg/kg/day for 7 days, orally.	------	**↓** MDA and NF-κB in the liver in a dose-dependent manner. **↑** SOD in the liver in a dose-dependent manner.	It reduces oxidative stress and inflammatory mediators in the liver.	Taştan Bal et al. [128]
Astaxanthin	Mice DHEA-induced PCOS	0.1 mg/kg, IP	↑ number of CL and Graafian follicles are observed.	**↓** ROS production and apoptosis in GC.	It reduces oxidative stress and consequently apoptosis of the CGs. It improves ovulatory capacity.	Ebrahimi et al. [129]
Astaxanthin + Curcumin (polyphenol)	Mice DHEA-induced PCOS	1.6 mg/day of astaxanthin + 0.1 mg/day of curcumin	**↓** T, E2, FSH, LH, AMH and the number of cysts. **↑** number of oocytes.	**↓** TC, ROS and IL-6.	It improves lipid metabolism, normalizes hormone levels, reduces oxidative stress and chronic inflammation, and improves ovulatory function ***.	Zhang et al. [130]

AMH, anti-Müllerian hormone; AR, androgen receptors; CL, corpora lutea; DHEA, dehydroepiandrosterone; E2, estradiol; EV, estradiol valerate; FSH, follicle-stimulating hormone; GC, granulosa cells; HDL, high-density lipoproteins; IL-6, interleukin 6; IP, intraperitoneal; LDL, low-density lipoproteins; LH, luteinizing hormone; MDA, malondialdehyde; NF-κB, nuclear factor kappa B; P4, progesterone; ROS, reactive oxygen species; SOD, superoxide dismutase; T, testosterone; TAC, total antioxidant capacity; TC, total cholesterol; TG, triglycerides; TNF-α, tumor necrosis factor-α. * Metformin (500 mg/kg/day, orally), an insulin sensitizing drug, has similar effects to lupeol treatment [117]. ** Flutamide (10 mg/kg/day), an antiandrogen, has similar effects to lupeol treatment [126]. *** The treatment is more effective when astaxanthin is combined with curcumin than when they are administered separately [130]. Downward arrows indicate decreases, and upward arrows indicate increases.

## Data Availability

No new data were created or analyzed in this study. Data sharing is not applicable to this article.
